# Granger Causality among Graphs and Application to Functional Brain Connectivity in Autism Spectrum Disorder

**DOI:** 10.3390/e23091204

**Published:** 2021-09-13

**Authors:** Adèle Helena Ribeiro, Maciel Calebe Vidal, João Ricardo Sato, André Fujita

**Affiliations:** 1 Data Science Institute, Columbia University, New York, NY 10027, USA; adele@cs.columbia.edu; 2 Insper Institute of Education and Research, São Paulo 04546-042, SP, Brazil; macielcv@insper.edu.br; 3 Center of Mathematics, Computing and Cognition, Universidade Federal do ABC, Santo André 09210-580, SP, Brazil; joao.sato@ufabc.edu.br; 4 Institute of Mathematics and Statistics, University of São Paulo, São Paulo 05508-090, SP, Brazil

**Keywords:** Granger causality, random graphs, spectral radius, brain connectivity, autism spectrum disorder

## Abstract

Graphs/networks have become a powerful analytical approach for data modeling. Besides, with the advances in sensor technology, dynamic time-evolving data have become more common. In this context, one point of interest is a better understanding of the information flow within and between networks. Thus, we aim to infer Granger causality (G-causality) between networks’ time series. In this case, the straightforward application of the well-established vector autoregressive model is not feasible. Consequently, we require a theoretical framework for modeling time-varying graphs. One possibility would be to consider a mathematical graph model with time-varying parameters (assumed to be random variables) that generates the network. Suppose we identify G-causality between the graph models’ parameters. In that case, we could use it to define a G-causality between graphs. Here, we show that even if the model is unknown, the spectral radius is a reasonable estimate of some random graph model parameters. We illustrate our proposal’s application to study the relationship between brain hemispheres of controls and children diagnosed with Autism Spectrum Disorder (ASD). We show that the G-causality intensity from the brain’s right to the left hemisphere is different between ASD and controls.

## 1. Introduction

Graphs have been extensively used to model high-dimensional systems with complex dependence structures. Networks are ubiquitous, from genes [1,2] to social systems [3,4]. Besides, with the advances in sensor technology, dynamic, time-evolving data have become more frequently available [5,6]. In this context, time-series analysis methods on dynamic networks became relevant to understand how networks evolve and interact. For example, we would like to infer the information flow between networks.

Clive Granger introduced a causality concept to analyze the relationships and influences among macroeconomic time series [7]. Granger causality consists of the idea that a cause cannot ever occur after its effect. To identify Granger causality (G-causality) between two time series, e.g., stock markets, we may use the vector autoregressive (VAR) model [8]. It is a well-established method and widely used in economy [9,10] and biology [11,12]. The VAR model has many variants. For example, the Dynamic VAR identifies time-varying G-causality [13,14,15]. The Sparse VAR is helpful when the number of parameters is greater than the number of observations [16,17,18]. The Nonlinear VAR identifies nonlinear G-causality [19,20] in contrast to the classic VAR that identifies only linear relationships. The Structural VAR allows an explicit structure of the contemporaneous effects and constraints on the lagged effects [21,22,23]. There is also an approach based on the canonical correlation analysis to identify G-causality between sets of time series [12,24]. However, in cases in which we are interested in identifying G-causality between networks, e.g., brain subnetworks, the straightforward application of a VAR model is not feasible. Indeed, they are objects composed of vertices and edges. Thus, a possibility would be to consider a mathematical model with parameters (assumed to be random variables) that generate the network.

Suppose we identify G-causality between the graph models’ parameters. In that case, we assume G-causality between two time series of graphs. However, in practice, the model that generates the empirical networks is rarely known. Additionally, model selection in complex and high-dimensional settings is difficult and comprises uncertainties.

Thus, the challenge consists of identifying a graph feature associated with the graph model’s parameters. Fujita et al. [25] suggested that the spectral radius is highly associated with the random graph model’s parameters (e.g., Erdös–Rényi, geometric, regular, Watts–Strogatz, and Barabási–Albert). Besides, they used it as a feature to infer the correlation between graphs. Based on this idea, we use the spectral radius to construct a VAR model for graphs. First, we evaluate our proposal’s performance in simulated data. Then, we illustrate its application to functional brain networks. We show that the Granger causality from the brain’s right to the left hemisphere is different between controls and children in Autism Spectrum Disorder.

## 2. Materials and Methods

### 2.1. Graph

A graph is an ordered pair G=(V,E), where *V* is a set of *n* vertices ($v_{1},v_{2},\ldots ,v_{n}$) and *E* is a set of *m* edges that connect two vertices of *V*. In this study, we will consider the case of graphs with a non-empty set of nodes and edges solely. Any undirected graph *G* with *n* vertices can be represented by its adjacency matrix $A^{G}$ with n×n elements $A_{ij}^{G}$(i,j=1,…,n); its value is $A_{ij}^{G}=A_{ji}^{G}=1$ if vertices $v_{i}$ and $v_{j}$ are connected and 0 otherwise. The spectrum of graph *G* is the set of eigenvalues of its adjacency matrix $A^{G}$. Since $A^{G}$ is symmetric, an undirected graph with *n* vertices has *n* real eigenvalues $\lambda_{1}\geq \lambda_{2}\geq \ldots \geq \lambda_{n}$.

### 2.2. Granger Causality between Graphs

Suppose we can predict the present and future values of $y_{t}$ better considering past values of $x_{t}$ than considering only past values of $y_{t}$. Then, we say that the time series $x_{t}$ Granger causes the time series $y_{t}$.

In this study, we want to identify Granger causality between two time series of graphs. To this end, we assume that random graph models generate the graphs. Additionally, we consider the parameters of the random graph models as random variables. We call the parameters of the distribution of this random variable as the hyperparameters of the graph models. Let Θ be the random variable that we will sample to generate the parameters of random graphs. The parameters determining the distribution of Θ are the hyperparameters of the random graphs. As an illustrative example, suppose that $G_{1}$ and $G_{2}$ are two Erdös–Rényi random graphs [26]. An Erdös–Rényi random graph has *n* labeled vertices, and we connect each pair of vertices by an edge with a given probability *p*. In this case, the probability *p* is the parameter of graph *G*. We describe the two time series of Erdös–Rényi random graphs as $G_{1}\left(p_{1t}\right)$ and $G_{2}\left(p_{2t}\right)$. Besides, we sample ($p_{1t}$) and ($p_{2t}$) from $\Theta_{1}$ and $\Theta_{2}$, respectively. We say that the random graph $G_{1}$ Granger causes $G_{2}$ if the random variable $\Theta_{1}$ Granger causes $\Theta_{2}$. Let ${\underset{\tilde{}}{\theta }}_{1}=\left\{\theta_{11},\theta_{12},\ldots ,\theta_{1T}\right\}$ and ${\underset{\tilde{}}{\theta }}_{2}=\left\{\theta_{21},\theta_{21},\ldots ,\theta_{2T}\right\}$ be two time series of size *T* from the random variables $\Theta_{1}$ and $\Theta_{2}$, respectively, and ${\underset{\tilde{}}{G}}_{1}\left({\underset{\tilde{}}{\theta }}_{1}\right)=\left\{G_{11}\left(\theta_{11}\right),G_{12}\left(\theta_{12}\right),\ldots ,G_{1T}\left(\theta_{1T}\right)\right\}$ and ${\underset{\tilde{}}{G}}_{2}\left({\underset{\tilde{}}{\theta }}_{2}\right)=\left\{G_{21}\left(\theta_{21}\right),G_{22}\left(\theta_{22}\right),\ldots ,G_{2T}\left(\theta_{2T}\right)\right\}$ be two time series of random graphs constructed by using ${\underset{\tilde{}}{\theta }}_{1}$ and ${\underset{\tilde{}}{\theta }}_{2}$, respectively. We describe the G-causality test between the random graphs $G_{1}$ and $G_{2}$, based on the samples ${\underset{\tilde{}}{G}}_{1}\left({\underset{\tilde{}}{\theta }}_{1}\right)$ and ${\underset{\tilde{}}{G}}_{2}\left({\underset{\tilde{}}{\theta }}_{2}\right)$ as follows. Let the null hypothesis be $H_{0}$: $\Theta_{1}$ *does not Granger cause* $\Theta_{2}$. Further, let the alternative hypothesis be $H_{1}$: $\Theta_{1}$ *Granger causes* $\Theta_{2}$. Suppose we know the graph models. Then, a straightforward way of identifying G-causality between $\Theta_{1}$ and $\Theta_{2}$ consists of estimating the parameters of the random graph models and then testing the absence of G-causality between them.

However, the graph model is rarely known for real-world graphs. Thus, the challenge consists of detecting G-causality only by observing the random graphs (and not the parameters). In other words, it is necessary to identify a feature of the graph that is highly associated with the parameters of the graph model. For several random graphs, we already know that the spectral radius (the largest eigenvalue—$\lambda_{1}$) is a function of the parameters of the graph model. For example, for the Erdös–Rényi random graph model, let *n* and *p* be the number of vertices and the probability that two vertices are connected by an edge, respectively. Then, the spectral radius of an Erdös–Rényi random graph is np. Another example is the regular random graphs. A regular random graph is a graph where each vertex has the same number of adjacent vertices. Let deg be the number of adjacent vertices; then, the spectral radius is deg.

Thus, considering that important structural and dynamical characteristics of a graph are defined by the parameters of the generating model, we can perform statistical analysis over graphs based solely on their spectral radii. Notably, ref. [25] already used the spectral radius to construct a framework to identify correlation between vectors of graphs. Thus, based on the same idea, we propose the use of the spectral radius to identify G-causality between time series of graphs. For simplicity, we will denote the spectral radius $\lambda_{1}$ just as λ.

### 2.3. Vector Autoregressive Model for Graphs

We often identify G-causality by fitting vector autoregressive (VAR) models. Consider the approach in which we represent the graphs by their spectral radii. Then, the extension of the VAR model for identifying G-causality among graphs is straightforward. Let:

- *k* be the number of time series of graphs;
- *p* be the order of the model (number of time points in the past to be analyzed);
- *T* be the length of the time series;
- $y_{i,t}$ be the spectral radius of the *i*th time series of graphs; and
- $\varepsilon_{i,t}$ be the vector of error terms for the *i*th graph, normally distributed, with zero mean and covariance matrix
  $$\Sigma =\left(\begin{array}{cccc} \sigma_{1,1}^{2} & \sigma_{2,1} & \ldots & \sigma_{k,1}\\ \sigma_{1,2} & \sigma_{2,2}^{2} & \ldots & \sigma_{k,2}\\ \vdots & \vdots & \ddots & \vdots \\ \sigma_{1,k} & \sigma_{2,k} & \ldots & \sigma_{k,k}^{2} \end{array}\right).$$

Note that the error terms $\varepsilon_{i,t}$ are serially uncorrelated, but may be contemporaneously correlated. In other words, Σ may not necessarily be an identity matrix.

Then, the equations system of a *k*-dimensional VAR model of order *p* is as follows:

  $\left\{\begin{array}{c} y_{1,t}=v_{1}+a_{1,1}^{1}y_{1,t-1}+\ldots +a_{1,1}^{p}y_{1,t-p}+\ldots +a_{k,1}^{1}y_{k,t-1}+\ldots +a_{k,1}^{p}y_{k,t-p}+\varepsilon_{1,t}\\ y_{2,t}=v_{2}+a_{1,2}^{1}y_{1,t-1}+\ldots +a_{1,2}^{p}y_{1,t-p}+\ldots +a_{k,2}^{1}y_{k,t-1}+\ldots +a_{k,2}^{p}y_{k,t-p}+\varepsilon_{2,t}\\ \vdots \\ y_{k,t}=v_{k}+a_{1,k}^{1}y_{1,t-1}+\ldots +a_{1,k}^{p}y_{1,t-p}+\ldots +a_{k,k}^{1}y_{k,t-1}+\ldots +a_{k,k}^{p}y_{k,t-p}+\varepsilon_{k,t} \end{array}\right.$

To simplify and facilitate the estimation of the coefficients of this model, we will rewrite the equations system in a matrix form.

Let

$$Y=\left(\begin{array}{cccc} y_{1,p+1} & y_{2,p+1} & \ldots & y_{k,p+1}\\ y_{1,p+2} & y_{2,p+2} & \ldots & y_{k,p+2}\\ \vdots & \vdots & \ddots & \vdots \\ y_{1,T} & y_{2,T} & \ldots & y_{k,T} \end{array}\right),$$

$$Z=\left(\begin{array}{ccccccccc} y_{1,p} & y_{1,p-1} & \ldots & y_{1,1} & \ldots & y_{k,p} & y_{k,p-1} & \ldots & y_{k,1}\\ y_{1,p+1} & y_{1,p} & \ldots & y_{1,2} & \ldots & y_{k,p+1} & y_{k,p} & \ldots & y_{k,2}\\ \vdots & \vdots & \ddots & \vdots & \ddots & \vdots & \vdots & \ddots & \vdots \\ y_{1,T-1} & y_{1,T-2} & \ldots & y_{1,T-p} & \ldots & y_{k,T-1} & y_{k,T-2} & \ldots & y_{k,T-p} \end{array}\right),$$

and

$$\beta =\left(\begin{array}{cccc} a_{1,1}^{1} & a_{1,2}^{1} & \ldots & a_{1,k}^{1}\\ \vdots & \vdots & \ddots & \vdots \\ a_{1,1}^{p} & a_{1,2}^{p} & \ldots & a_{1,k}^{p}\\ \vdots & \vdots & \ddots & \vdots \\ a_{k,1}^{1} & a_{k,2}^{1} & \ldots & a_{k,k}^{1}\\ \vdots & \vdots & \ddots & \vdots \\ a_{k,1}^{p} & a_{k,2}^{p} & \ldots & a_{k,k}^{p} \end{array}\right).$$

Then, the VAR model can be written in a matrix form as:

(1) Y=Zβ+u.

The coefficients of the model, $a_{i,j}^{l}$, with i,j=1,…,k and l=1,…,p, can be estimated by Ordinary Least Squares (OLS) as

(2) $$\hat{\beta }={\left(Z^{\prime }Z\right)}^{-1}Z^{\prime }Y.$$

The ((T−p)×k) matrix of residuals $\hat{u}$ can be obtained as

(3) $$\hat{u}=Y-Z\hat{\beta },$$

and the (k×k) covariance matrix Σ as

(4) $$\hat{\Sigma }=\frac{{\hat{u}}^{\prime }\hat{u}}{\left(T-p)-(kp\right)}.$$

### 2.4. Statistical Tests

Assume that a graph time series is linearly associated with any lagged version of itself and the other graph. Then, a necessary and sufficient condition for graph time series $y_{i,t}$ being not Granger-causal for graph time series $y_{j,t}$ is that $a_{i,j}^{l}=0$, for l=1,…,p. Thus, we may identify Granger non-causality by testing the significance of the entries $a_{i,j}^{l}$ of the matrix of autoregressive coefficients (β) of the VAR model.

The hypothesis test of connectivity significance β is $H_{0}:C\beta =0$ versus $H_{1}:C\beta \neq 0$, where C is a matrix of contrasts for the parameters we are interested in. We can achieve this test by applying Wald’s test [27] (Section 2.4.1) or a parametric bootstrap procedure (Section 2.4.2).

#### 2.4.1. Wald’s Test

Suppose we are interested in testing whether $y_{i,t}$ Granger causes $y_{j,t}$. Let c be a (1×k) matrix with one in the *i*th position and zero in the other positions, and let 0 be a (1×k) matrix of zeros. Then, we define the (p×(kp)) matrix of contrasts C as:   

$$C=\left(\begin{array}{cccc} c & 0 & \ldots & 0\\ 0 & c & \ldots & 0\\ \vdots & \vdots & \ddots & \vdots \\ 0 & 0 & \ldots & c \end{array}\right).$$

Let ${\hat{\beta }}_{j}=\left({\hat{a}}_{1,j}^{1},\ldots ,{\hat{a}}_{1,j}^{p},\ldots ,{\hat{a}}_{k,j}^{1},\ldots ,{\hat{a}}_{k,j}^{p}\right)$ be the kp×1 vector with the estimates of the autoregressive coefficients for $y_{j,t}$. Further, let ${\hat{\Sigma }}_{j,j}$ be the *j*th column of the *j*th row of the estimated covariance matrix $\hat{\Sigma }$. Then, Wald’s test statistic is defined as follows:

(5) $$W=\frac{{\left(C{\hat{\beta }}_{j}\right)}^{\prime }{\left(C{\left(Z^{\prime }Z\right)}^{-1}C^{\prime }\right)}^{-1}\left(C{\hat{\beta }}_{j}\right)}{{\hat{\Sigma }}_{j,j}}.$$

Under the null hypothesis that $C\beta_{j}=0$, Wald’s test statistic *W* follows a $\chi^{2}$ distribution with rank(C) degrees of freedom.

#### 2.4.2. Bootstrap Procedure

When the time series length is limited, such as functional magnetic resonance imaging (fMRI) data, Wald’s test assumption (T→∞) does not hold anymore. Then, we suggest the use of the following parametric bootstrap algorithm:

1. Fit the VAR model (Equation (1)).
2. Estimate both the VAR model coefficients (Equation (2)) and residuals (Equation (3)).
3. Resample with replacement the residuals obtained in step 2.
4. To test the G-causality from graph $y_{i,t}$ to graph $y_{j,t}$, estimate Wald’s test statistic *W* (Equation (5)). Then, construct a model under the null hypothesis, i.e., assume a model where the VAR coefficients $a_{ij}^{l}=0$ ∀l=1,…,p. The other coefficients remain as initially estimated in step 2.
5. Resample the residuals obtained in step 2 and use the model specified in step 4 to simulate a bootstrap multivariate time series.
6. Estimate the coefficients $a_{i,j}^{l*}$ of the bootstrap time series obtained in step 5 and calculate Wald’s test statistic $W^{*}$.
7. Go to step 3 until you obtain the desired number of bootstraps.
8. Estimate the *p*-value by calculating the fraction of replicates of $W^{*}$ on the bootstrap dataset, which is at least as large as the observed statistic *W* on the original dataset.

### 2.5. Random Graph Models

Here we describe some examples of random graph models that we will use in our simulation study.

#### 2.5.1. Erdös–Rényi Random Graph

Erdös–Rényi random graphs [26] are one of the most studied random graphs. Erdös and Rényi defined a random graph as *n* labeled vertices. We connect each pair of vertices by an edge with a given probability *p*.

The spectral radius of an Erdös–Rényi random graph is np [28].

We used the function erdos.renyi.game of the R package igraph to generate Erdös–Rényi random graphs. We downloaded the igraph package version 1.2.4 from the R website (http://www.r-project.org, accessed on 13 February 2019).

#### 2.5.2. Geometric Random Graph

A geometric random graph (GRG) is a spatial network. We construct an undirected graph by randomly placing *n* vertices in some topological space $R^{d}$ according to a probability distribution (e.g., uniform distribution). Then, we connect two vertices by an edge if their distance is smaller than a neighborhood radius *r*.

The spectral radius of a GRG converges almost surely to $r^{d}$ [29].

We used the function grg.game of the R package igraph to generate geometric random graphs.

#### 2.5.3. Regular Random Graph

A regular random graph is a graph where each vertex has the same number of adjacent vertices, i.e., every vertex has the same degree. A regular random graph with vertices of degree deg is called a deg-regular graph or regular graph of degree deg [30].

Regular random graphs of degree deg=0,1,2,3 are well known:

- a 0-regular graph consists of disconnected vertices;
- a 1-regular graph consists of disconnected edges;
- a 2-regular graph consists of disconnected cycles and infinite chains;
- a 3-regular graph is known as a cubic graph.

The spectral radius of a deg-regular graph is deg [31].

We used the function k.regular.game of the R package igraph to generate regular random graphs.

#### 2.5.4. Watts–Strogatz Random Graph

The Watts–Strogatz random graph [32] presents small-world properties and a higher clustering coefficient than Erdös–Rényi random graphs. redThe construction of a Watts–Strogatz random graph depends on three parameters: the number of vertices *n*, the number of neighbors (mean degree) nei, and the rewiring probability $p_{w}$. We start by constructing a ring with *n* vertices. Then, we connect every vertex to its first nei neighbors ($\frac{nei}{2}$ on either side. For each vertex in the ring, we reconnect with probability $p_{w}$ the edge that connects it to its nearest neighbor to a vertex chosen uniformly at random over the entire ring. We do this process moving clockwise around the ring until completing one lap. Next, we consider the edges that connect the vertices to their second-nearest neighbors clockwise. As in the previous step, we randomly rewire each edge with probability $p_{w}$. We continue this process circulating the ring and proceeding outward to more distant neighbors after each lap until each edge in the original lattice has been considered once.

To the best of our knowledge, the spectral radius of a Watts–Strogatz random graph is not analytically defined. However, there is empirical evidence that it is a function of $p_{w}$ and nei [33].

We used the function watts.strogatz.game of the R package igraph to generate Watts–Strogatz random graphs.

#### 2.5.5. Barabási–Albert Random Graph

Barabási–Albert random graphs have a power-law degree distribution [34]. It is due to the vertices’ preferential attachment, i.e., the more connected a vertex is, the more likely it is to receive new edges [34]. proposed the following construction. Start with a small number of ($n_{0}$) vertices. At every time step, add a new vertex with ($m_{1}\leq n_{0}$) edges that connect the new vertex to $m_{1}$ different vertices already present in the system. When choosing the vertices to which the new vertex connects, assume that the probability of connecting a new vertex to the vertex $v_{i}$ is proportional to the degree of the vertex $v_{i}$ and the scaling exponent $p_{s}$ ($P\left(v_{i}\right)∼deg{\left(v_{i}\right)}^{p_{s}}$, where $deg(v_{i})$ is the degree of the vertex $v_{i}$ in the current time step) which indicates the proportionality order ($p_{s}=1$ linear; $p_{s}=2$ quadratic and so on).

Let $k_{0}$ be the smallest degree. Then, the spectral radius of the Barabási–Albert random graph is of the order of $k_{0}^{1/2}n^{1/2(p_{s}-1)}$ [35].

We used the function barabasi.game of the R package igraph to generate Barabási–Albert random graphs.

### 2.6. Simulation Study

We evaluated the performance of our proposal by simulation studies. Next, we describe five different scenarios. The error terms $\varepsilon_{i,t}$ are normal, centered at zero, and weakly correlated, i.e., $\mathrm{Cov}(\varepsilon_{i,t},\varepsilon_{j,t})=0.1$ if i≠j, and 1 if i=j.

**Scenario** **1:** data were generated by the following model where $y_{1,t}$ and $y_{2,t}$ are not Granger causally dependent:
  $$\left\{\begin{array}{c} y_{1,t}=0.5\;y_{1,t-1}+\varepsilon_{1,t}\\ y_{2,t}=0.5\;y_{2,t-1}+\varepsilon_{2,t} \end{array}\right.$$
**Scenario** **2:** data were generated by the following model involving a direct Granger causal effect from $y_{1,t}$ to $y_{2,t}$:
  $$\left\{\begin{array}{c} y_{1,t}=0.5\;y_{1,t-1}+\varepsilon_{1,t}\\ y_{2,t}=0.5\;y_{1,t-1}+\varepsilon_{2,t} \end{array}\right.$$
**Scenario** **3:** data were generated by the following model where $y_{1,t}$ Granger causes both $y_{2,t}$ and $y_{3,t}$:
  $$\left\{\begin{array}{c} y_{1,t}=\varepsilon_{1,t}\\ y_{2,t}=0.5\;y_{1,t-1}+\varepsilon_{2,t}\\ y_{3,t}=-0.5\;y_{1,t-1}+\varepsilon_{3,t} \end{array}\right.$$
**Scenario** **4:** data were generated by a model involving direct and indirect Granger causal effects (1) $y_{1,t}\to y_{2,t}$; (2) $y_{2,t}\to y_{3,t}$, (3) $y_{1,t}\to y_{3,t}$, and (4) $y_{3,t}\to y_{4,t}$, as follows:
  $$\left\{\begin{array}{c} y_{1,t}=\varepsilon_{1,t}\\ y_{2,t}=0.5\;y_{1,t-1}+\varepsilon_{2,t}\\ y_{3,t}=-0.5\;y_{1,t-2}+0.5\;y_{2,t-1}+\varepsilon_{3,t}\\ y_{4,t}=0.5\;y_{3,t-1}+\varepsilon_{4,t}. \end{array}\right.$$
**Scenario** **5:** data were generated by the following model with a feedback loop $\left(y_{1,t}\to y_{2,t}\to y_{3,t}\to y_{4,t}\to y_{2,t}\right)$:
  $$\left\{\begin{array}{c} y_{1,t}=\varepsilon_{1,t}\\ y_{2,t}=0.5\;y_{1,t-1}-0.5\;y_{4,t-1}+\varepsilon_{2,t}\\ y_{3,t}=-0.5\;y_{2,t-2}+\varepsilon_{3,t}\\ y_{4,t}=0.5\;y_{3,t-1}+\varepsilon_{4,t}. \end{array}\right.$$

We normalized the time series obtained in scenarios 1 to 5 to the interval [0;1] using the inverse-logit function. Then, we used them as parameters of the random graph models as follows:

- Erdös-Rényi random graph: values corresponded to the probability *p* of two vertices being connected.
- Random geometric graph: values corresponded to the neighborhood radius parameter, *r*.
- Random regular graph: the integer part of the values after being multiplied by 10 corresponded to the deg.
- Watts–Strogatz random graph: values corresponded to the rewiring probability, $p_{w}$.
- Barabási–Albert random graph: values, after being multiplied by two, corresponded to the power of the preferential attachment.

For the Watts–Strogatz random graph model, we set the number of neighbors nei=3. For the Barabási–Albert random graph model, we set the number of edges to be included at each iteration to one.

We simulated all graphs using the R package igraph. We considered different numbers of vertices (n=60,90,120,150,200,300) and time series length (T=25,50,75,100). We repeated each setting 1 000 times.

### 2.7. Application

The Autism Spectrum Disorder (ASD) etiology is complex and not completely understood [36]. It involves several risk factors, such as genetic, environmental, psychological, and neurobiological [37,38]. Thus, a multidisciplinary group composed of physicians and psychologists usually diagnoses it through clinical interviews and tests. Then, they identify a combination of unusual behavioral characteristics, such as assessing deficits in social communication, social reciprocity, and repetitive and stereotyped behaviors and interests [39]. These symptoms frequently manifest during the first 3 years of life. They usually come with developmental differences in brain anatomy, functioning, and functional brain connectivity. Current studies suggest that ASD is a brain systems disorder [40,41,42,43]. Additionally, anatomical abnormalities are subtle but widespread over the brain [44]. Thus, one straightforward approach to enhancing our comprehension of this disorder’s neural substrates is to investigate differences in brain connectivity compared to controls. Most studies focus on finding differences between region-to-region functional connectivity or network centrality measures. Due to the lack of a suitable methodological framework, investigations of how the structural organization in one brain sub-network is associated with another are limited. Moreover, the description of these “networks of networks” in clinical populations remains unexplored. Here we establish a novel framework to identify how the information flow (Granger causality) between the left and right hemispheres of the brain changes between controls and ASD.

#### 2.7.1. ABIDE I Dataset

**Figure 1:**
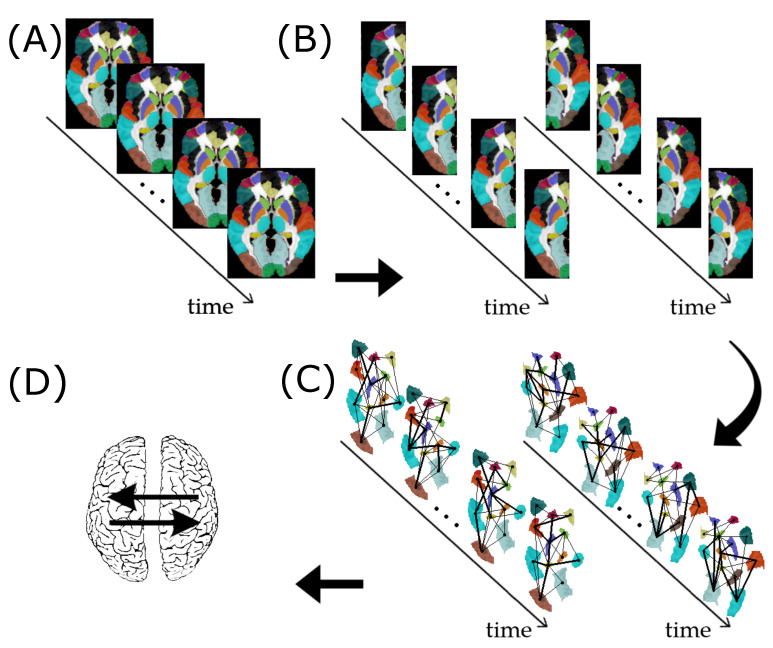
Resting-state fMRI data preprocessing for assessing subject-specific G-causality between the left and right brain hemispheres. (**A**) fMRI data segmented into 90 ROIs according to the AAL atlas. (**B**) Separation of the ROIs as belonging to the left or right hemisphere. (**C**) Construction of the functional brain networks time series for the left and right hemispheres. (**D**) Identification of G-causality between the left and right hemispheres.

We downloaded 1112 individuals’ resting-state fMRI data from the ABIDE Consortium website (http://fcon_1000.projects.nitrc.org/indi/abide/, accessed on 18 January 2018). The ABIDE dataset was fully anonymized in compliance with the HIPAA Privacy Rules and the 1000 Functional Connectomes Project/INDI protocols. Protected health information was not included in this dataset. Further details are available at the ABIDE Consortium website. We performed the pre-processing of the fMRI data using the Athena pipeline (http://www.nitrc.org/plugins/mwiki/index.php/neurobureau:AthenaPipeline, accessed on January 18th, 2018). We defined the 116 regions of interest (ROIs) using the Anatomical Automatic Labeling (AAL) brain atlas [45]. Then, we excluded 26 cerebellar ROIs. We labeled the remaining 90 ROIs as part of the left or right hemisphere according to the side containing the most number of voxels (Figure 1A). Then, we obtained 45 regions in each hemisphere. In other words, we represented each hemisphere as a network composed of 45 vertices. We considered the average time series within the ROIs as the region’s representatives (Figure 1B). To minimize head movement effects, we excluded subjects with mean framewise displacement (FD) greater than 0.2. This process resulted in the inclusion of 737 subjects (429 controls and 308 individuals diagnosed with ASD) for subsequent analyses. Thus, the dataset used in this study comprises 429 controls (340 males, mean age 17.26±7.62) and 308 ASD (270 males, 17.72±8.24 years).

#### 2.7.2. Granger Causality Analysis

A typical procedure for constructing functional brain networks (FBNs) is the Pearson correlation. Since we are interested in the dynamics of the FBNs, we calculated a time-varying Pearson correlation for each time point. The strategy is similar to the one described by [14]. However, instead of using a wavelet-based approach, for simplicity, we used splines. Thus, we obtained two undirected graphs per individual and per time point: one for the left and another for the right hemispheres of the brain. The vertices represent the ROIs. The edge weights represent the Pearson correlation coefficients among ROIs (Figure 1C).

We hypothesize that the brain hemispheres interact differently between controls and ASD. To test this hypothesis, first, we applied the proposed VAR method for graphs to identify G-causality between the left and right brain hemispheres networks. This analysis was performed separately for each sampled individual, using the same VAR’s order *p*, estimated by AIC. To infer G-causality from the left to the right brain hemisphere, we obtained Wald’s test statistics $W_{\mathrm{Left}\to \mathrm{Right}}$ associated with the null hypothesis that the autoregressive coefficients $a_{\mathrm{Left},\mathrm{Right}}^{l}=0$, for l=1,…,p. Similarly, to infer G-causality from the right to the left brain hemisphere, we obtained Wald’s test statistics $W_{\mathrm{Right}\to \mathrm{Left}}$ associated with the null hypothesis that the autoregressive coefficients $a_{\mathrm{Right},\mathrm{Left}}^{l}=0$, for l=1,…,p. See Figure 1D.

To determine whether the G-causality intensity between the brain hemispheres was different in autistic subjects, we linearly regressed the Box–Cox transformed Wald’s test statistics, previously computed for all sampled individuals, on the main effect of FD, and the main and interaction effects of SEX (0: male, 1: female), AGE, and ASD diagnosis (0: control, 1: ASD). Since we had two Wald’s test statistics, one for each causality direction, we carried out two independent linear regressions. The Box–Cox transformation made the distribution of Wald’s test statistics approximately Gaussian. To control the site’s effects, we fitted a linear mixed model with two components of variance: $\gamma_{\mathrm{SITE}}$, for modeling the variability between sites, and ε, for capturing the residual variability. We assumed both random effects were independent and normally distributed. Specifically, let $W_{\mathrm{Right}\to \mathrm{Left}}$ be Wald’s test statistic obtained for assessing the causality from the right to the left brain hemisphere and consider an appropriate value for the parameter κ of the Box–Cox transformation. For example, one may consider the parameter κ that maximizes the model’s log-likelihood with all covariates of interest. Additionally, let $\beta_{\mathrm{FD}}$, $\beta_{\mathrm{SEX}}$, $\beta_{\mathrm{AGE}}$, and $\beta_{\mathrm{ASD}}$ be the coefficients for the main effects of FD, SEX, AGE, and ASD diagnosis, respectively. Furthermore, let $\beta_{\mathrm{SEX}\times \mathrm{AGE}}$, $\beta_{\mathrm{SEX}\times \mathrm{ASD}}$, $\beta_{\mathrm{AGE}\times \mathrm{ASD}}$, and $\beta_{\mathrm{SEX}\times \mathrm{AGE}\times \mathrm{ASD}}$ be the coefficients for the interaction effects involving SEX, AGE, and ASD diagnosis. Then, we considered the following linear mixed model:

(6) $$\begin{array}{cc} \; & \frac{W_{\mathrm{Right}\to \mathrm{Left}}^{\kappa }-1}{\kappa }=\alpha +\beta_{\mathrm{FD}}\mathrm{FD}+\beta_{\mathrm{SEX}}\mathrm{SEX}+\beta_{\mathrm{AGE}}\mathrm{AGE}+\beta_{\mathrm{ASD}}\mathrm{ASD}+\\ \; & \;\beta_{\mathrm{SEX}\times \mathrm{AGE}}\mathrm{SEX}\times \mathrm{AGE}+\beta_{\mathrm{AGE}\times \mathrm{ASD}}\mathrm{AGE}\times \mathrm{ASD}+\beta_{\mathrm{SEX}\times \mathrm{ASD}}\mathrm{SEX}\times \mathrm{ASD}+\\ \; & \;\beta_{\mathrm{SEX}\times \mathrm{AGE}\times \mathrm{ASD}}\mathrm{SEX}\times \mathrm{AGE}\times \mathrm{ASD}+\gamma_{\mathrm{SITE}}+\varepsilon \end{array}$$

We considered an analogous model for Wald’s test statistic for assessing the G-causality from the left to the right brain hemisphere.

## 3. Results and Discussions

### 3.1. Simulation Study

To evaluate the control of the rate of false positives and the power of the proposed method, we simulated scenarios 1 to 5 as described in Section 2.6. Then, we constructed receiver operating characteristic (ROC) curves. We considered different times series lengths (T=25,50,75,100) and graphs sizes (n=60,90,120,150,200,300). We repeated each setting 1000 times. We set the number of bootstrap replicates to 1000.

**Figure 2:**
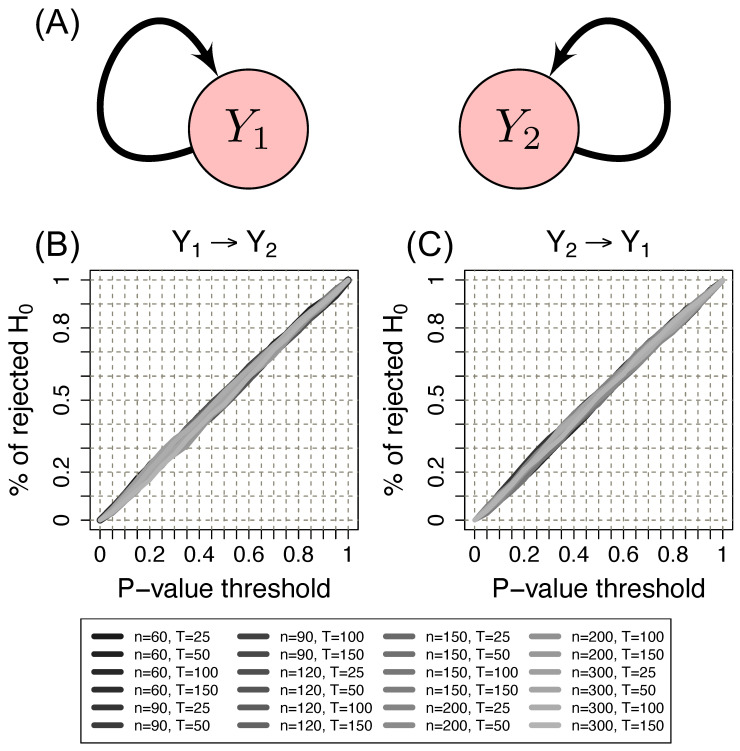
(**A**) Schema of scenario 1. Two time series of Erdös–Rényi random graphs ($Y_{1}$ and $Y_{2}$) without G-causality between them. (**B**) ROC curve to evaluate the control of the type I error from $Y_{1}$ to $Y_{2}$. (**C**) ROC curve to evaluate the control of the type I error from $Y_{2}$ to $Y_{1}$. The *x*-axis represents the *p*-value threshold. The *y*-axis represents the proportion of rejected null hypotheses given a *p*-value threshold. *n*: the number of vertices. *T*: the time series length. Note that the proportion of identified Granger causalities under the null hypothesis is as expected by the *p*-value threshold (ROC curves lie at the diagonal). Therefore the proposed method indeed controls the type I error.

Figure 2 shows the ROC curves for scenario 1 (under the null hypothesis, i.e., no G-causality between the time series) using the Erdös–Rényi random graph model. Results using other random graph models are similar. The *x*-axis represents the *p*-value threshold. The *y*-axis represents the proportion of rejected null hypotheses given a *p*-value threshold. Under the null hypothesis, we expected that the ROC curve lay at the diagonal. We observed that the proposed method indeed controlled the type I error (all ROC curves indeed lay at the diagonal).

**Figure 3:**
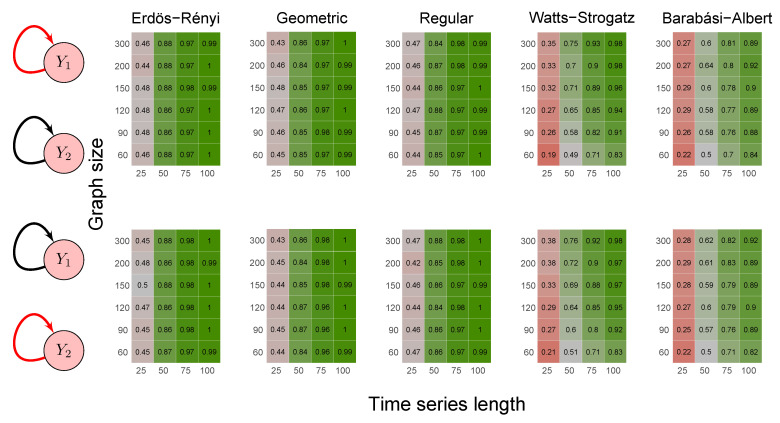
Heatmaps represent the proportion of rejected null hypotheses at a *p*-value threshold of 0.05. At the left, we show the direction of G-causality (direction of the edges) in Scenario 1. Heatmaps on the right side represent the proportion of rejected null hypotheses highlighted in red in the left schema. The four columns of each heatmap correspond to the results obtained by varying the time series length T=25,50,75,100. The six rows correspond to the results obtained by varying the sizes of the graphs (number of vertices) n=60,90,120,150,200,300. The “greener” the heatmap is, the greater is the power of the test. In contrast, the “redder” it is, the lower is the proportion of rejected null hypotheses. We simulated five random graph models, namely Erdös–Rényi, geometric, regular, Watts–Strogatz, and Barabási–Albert.

**Figure 4:**
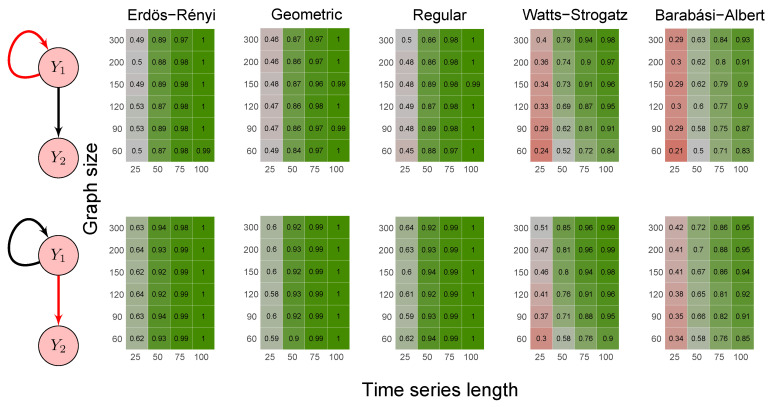
Heatmaps represent the proportion of rejected null hypotheses at a *p*-value threshold of 0.05. At the left, we show the direction of G-causality (direction of the edges) in Scenario 2. Heatmaps on the right side represent the proportion of rejected null hypotheses highlighted in red in the left schema. The four columns of each heatmap correspond to the results obtained by varying the time series length T=25,50,75,100. The six rows correspond to the results obtained by varying the sizes of the graphs (number of vertices) n=60,90,120,150,200,300. The “greener” the heatmap is, the greater is the power of the test. In contrast, the “redder” it is, the lower is the proportion of rejected null hypotheses. We simulated five random graph models, namely Erdös–Rényi, geometric, regular, Watts–Strogatz, and Barabási–Albert.

**Figure 5:**
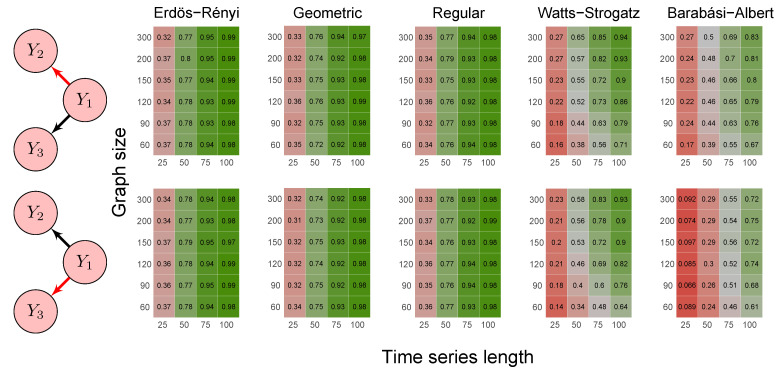
Heatmaps represent the proportion of rejected null hypotheses at a *p*-value threshold of 0.05. At the left, we show the direction of G-causality (direction of the edges) in Scenario 3. Heatmaps on the right side represent the proportion of rejected null hypotheses highlighted in red in the left schema. The four columns of each heatmap correspond to the results obtained by varying the time series length T=25,50,75,100. The six rows correspond to the results obtained by varying the sizes of the graphs (number of vertices) n=60,90,120,150,200,300. The “greener” the heatmap is, the greater is the power of the test. In contrast, the “redder” it is, the lower is the proportion of rejected null hypotheses. We simulated five random graph models, namely Erdös–Rényi, geometric, regular, Watts–Strogatz, and Barabási–Albert.

**Figure 6:**
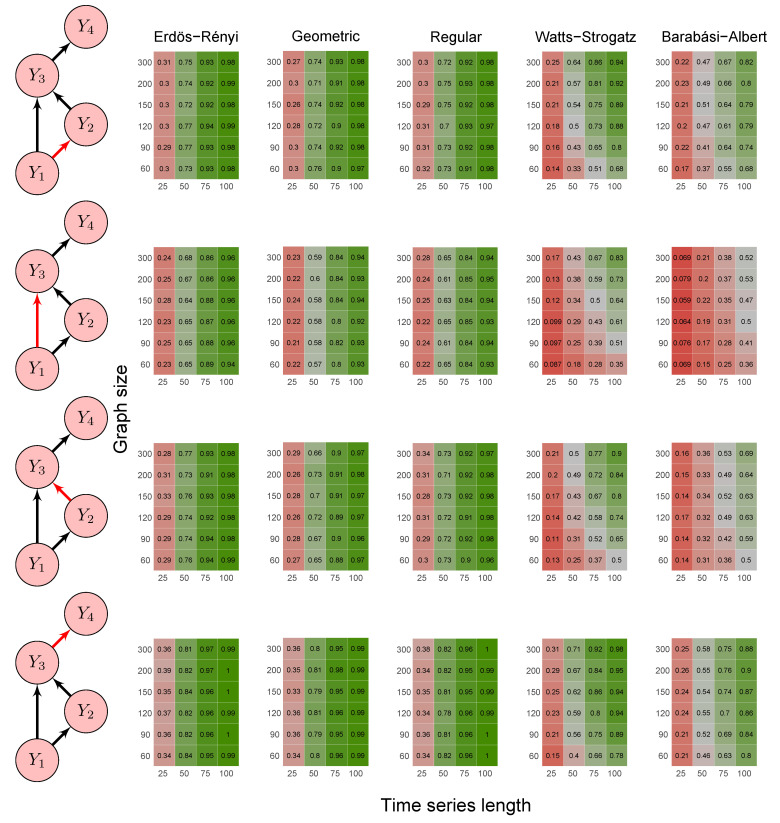
Heatmaps represent the proportion of rejected null hypotheses at a *p*-value threshold of 0.05. At the left, we show the direction of G-causality (direction of the edges) in Scenario 4. Heatmaps on the right side represent the proportion of rejected null hypotheses highlighted in red in the left schema. The four columns of each heatmap correspond to the results obtained by varying the time series length T=25,50,75,100. The six rows correspond to the results obtained by varying the sizes of the graphs (number of vertices) n=60,90,120,150,200,300. The “greener” the heatmap is, the greater is the power of the test. In contrast, the “redder” it is, the lower is the proportion of rejected null hypotheses. We simulated five random graph models, namely Erdös–Rényi, geometric, regular, Watts–Strogatz, and Barabási–Albert.

**Figure 7:**
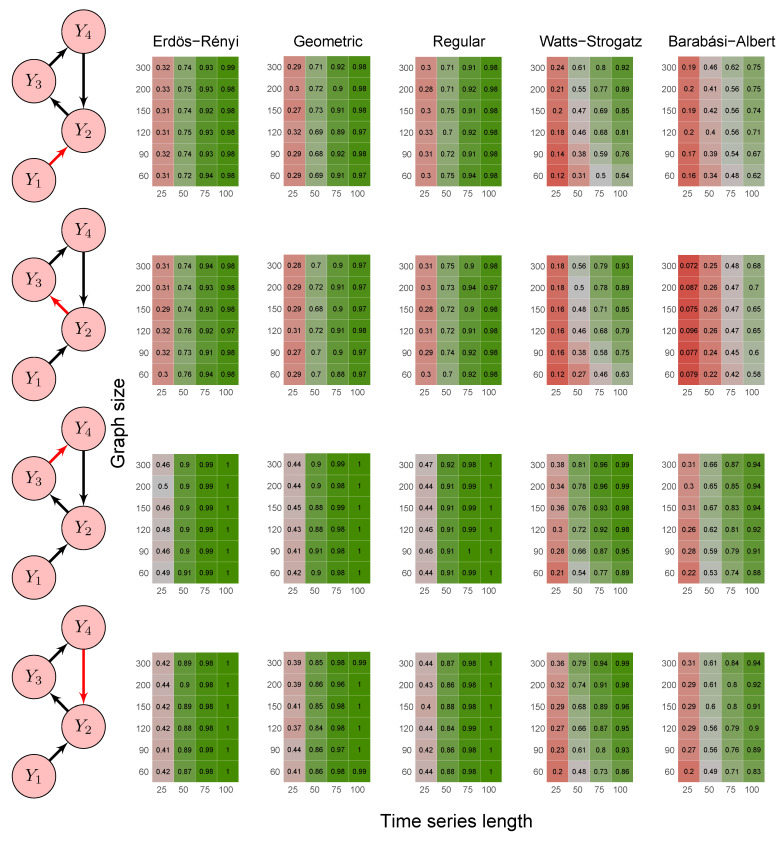
Heatmaps represent the proportion of rejected null hypotheses at a *p*-value threshold of 0.05. At the left, we show the direction of G-causality (direction of the edges) in Scenario 5. Heatmaps on the right side represent the proportion of rejected null hypotheses highlighted in red in the left schema. The four columns of each heatmap correspond to the results obtained by varying the time series length T=25,50,75,100. The six rows correspond to the results obtained by varying the sizes of the graphs (number of vertices) n=60,90,120,150,200,300. The “greener” the heatmap is, the greater is the power of the test. In contrast, the “redder” it is, the lower is the proportion of rejected null hypotheses. We simulated five random graph models, namely Erdös–Rényi, geometric, regular, Watts–Strogatz, and Barabási–Albert.

To evaluate the method’s power, we carried out the simulations described in scenarios 2 to 5 (Section 2.6) using five random graph models, namely Erdös–Rényi, geometric, regular, Watts–Strogatz, and Barabási–Albert. We set the *p*-value threshold to 0.05. We summarize the results in the heatmaps of Figure 3, Figure 4, Figure 5, Figure 6 and Figure 7. The “greener” the heatmap is, the greater was the proportion of rejected null hypotheses. In contrast, the “redder” it is, the lower was the power.

First, it is possible to notice that the power of the test was more remarkable as the time series length increased. Moreover, for the Watts–Strogatz and Barabási–Albert random graph models, the power of the test was also higher as the number of vertices of the graph increased. Therefore, we noticed that these two random graph models required greater graph sizes to obtain better estimates of the spectral radii. In addition, we confirmed that the method could identify G-causality in different structures, such as in the presence of a mediator (Figure 6) and loop (Figure 7).

**Figure 8:**
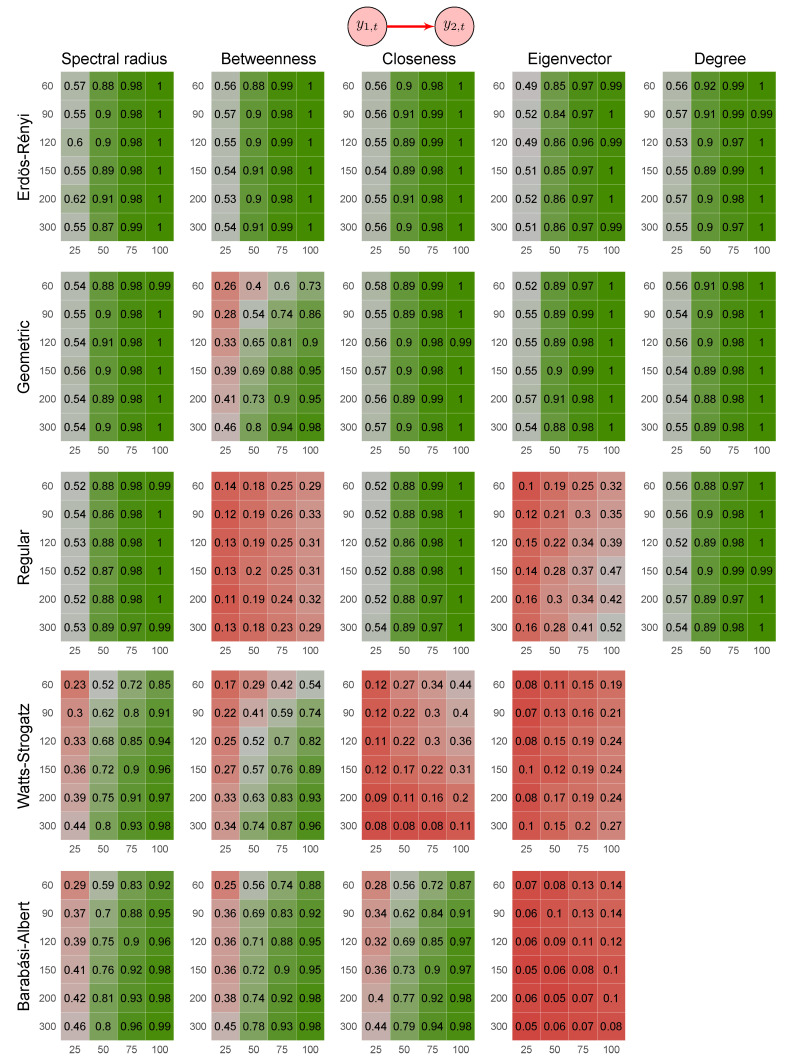
Heatmaps represent the proportion of rejected null hypotheses at a *p*-value threshold of 0.05. At the top, the direction of G-causality (direction of the edge) in Scenario 2. Each cell has varying colors from red to green representing the statistical power (proportion of rejected null hypotheses). The four columns of each heatmap correspond to the results obtained by varying the time series length T=25,50,75,100. The six rows correspond to the results obtained by varying the sizes of the graphs (number of vertices) n=60,90,120,150,200,300. The “greener” the heatmap is, the greater is the power of the test. In contrast, the “redder” it is, the lower is the proportion of rejected null hypotheses. We simulated five random graph models, namely Erdös–Rényi, geometric, regular, Watts–Strogatz, and Barabási–Albert. We do not show the heatmaps for Watts–Strogatz and Barabási–Albert random graph models because alterations in the parameter do not alter the degree.

One may consider using another graph feature instead of the spectral radius, such as one of the centrality measures (e.g., betweenness, closeness, eigenvector, and degree). Thus, we repeated the analysis by using these other features. Figure 8 shows the heatmaps describing the results of these simulations. By analyzing Figure 8, we notice that the power of the proposed method based on the spectral radius was greater (or at least equivalent) than when based on other features.

### 3.2. Application

**Figure 9:**
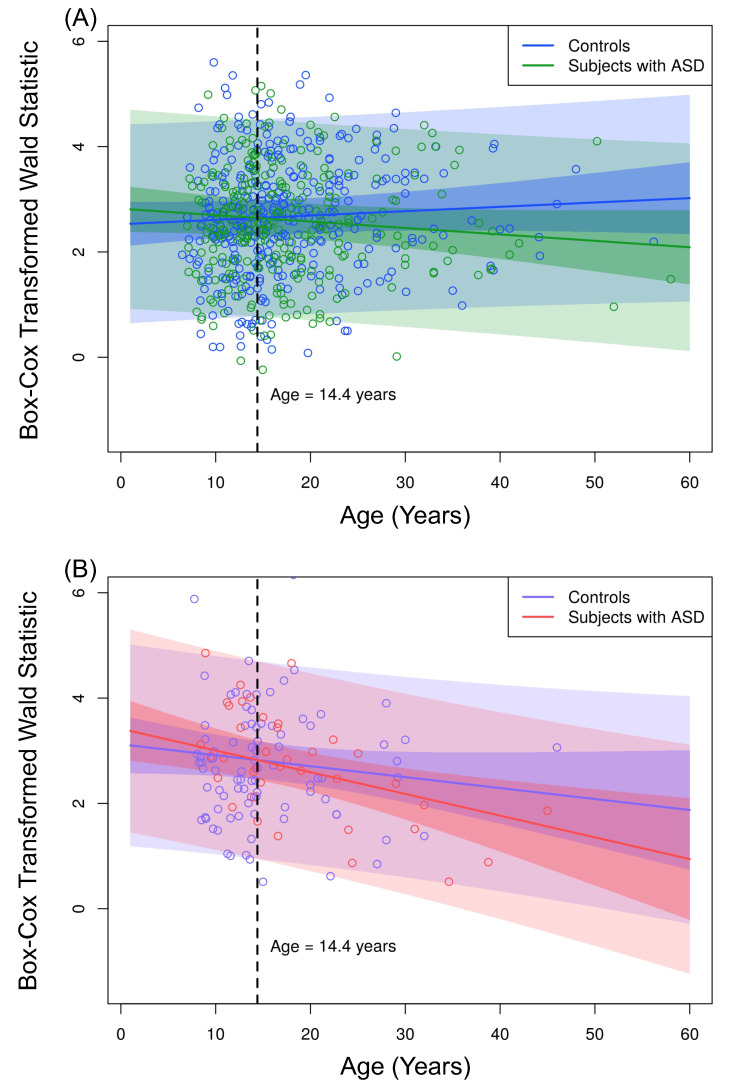
Observed values of the Box–Cox transformed Wald’s test statistic $W_{\mathrm{Right}\to \mathrm{Left}}$ obtained from the VAR method with order five. (**A**) Male controls (blue dots) and male subjects with ASD (green dots). (**B**) Female controls (violet dots) and female subjects with ASD (pink dots). We fitted the curves considering Equation (6) with FD = 0 and ages from 0 to 60 years. Narrow shaded areas represent the 95% prediction intervals considering only the standard deviation of the random effect $\gamma_{\mathrm{SITE}}$. The broad shaded areas represent the 95% prediction intervals considering the standard deviation of the error ε.

We estimated the VAR order as five for all sampled individuals by using the Akaike Information Criterion (AIC). Considering a significance level of 5%, the G-causality from the left to the right brain hemispheres is not significantly different between ASD and control groups. Additionally, all other effects were considered non-significant by fitting the mixed model shown in Equation (6). However, as shown in Table 1, we identified a differential G-causality from the right to the left brain hemispheres in ASD. By using Equation (6), we identified a significant interaction effect between AGE and ASD diagnosis ($\beta_{\mathrm{AGE}\times \mathrm{ASD}}=-0.020$, p=0.022). Besides, we identified a significant interaction effect between AGE and SEX ($\beta_{\mathrm{AGE}\times \mathrm{SEX}}=-0.029$, p=0.021). Figure 9 panels A and B illustrate the interaction effect between AGE and ASD, separately for male and female subjects, because of the significant interaction effect between AGE and SEX.

The loss of functional connectivity from the right to the left brain hemisphere as age increased was significantly higher in subjects with autism. In other words, the G-causality significantly decreased 0.012 ($\beta_{\mathrm{AGE}\times \mathrm{ASD}}-\beta_{\mathrm{AGE}}=0.020-0.008$) each year in autistic male subjects. In contrast, we did not identify significant changes in male controls ($\beta_{\mathrm{AGE}}=0.008$, p=0.261). We identified a a decrease of 0.041 ($\beta_{\mathrm{AGE}\times \mathrm{ASD}}+\beta_{\mathrm{AGE}\times \mathrm{SEX}}-\beta_{\mathrm{AGE}}=0.020+0.029-0.008$) in subjects with autism and 0.021 ($\beta_{\mathrm{AGE}\times \mathrm{SEX}}-\beta_{\mathrm{AGE}}=0.029-0.008$) each year in controls by analyzing females.

**Figure 10:**
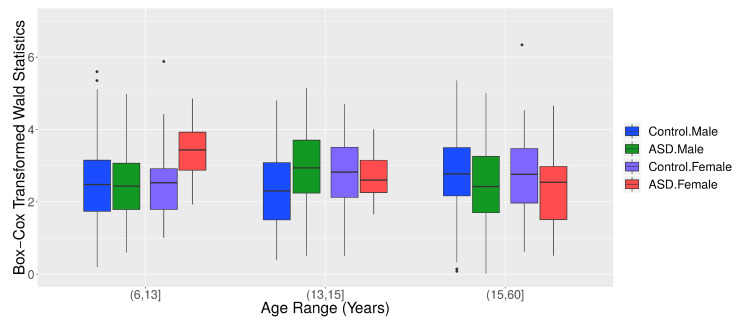
Boxplots of the Box–Cox transformed Wald’s test statistic $W_{\mathrm{Right}\to \mathrm{Left}}$ obtained from the VAR method for graphs, separated by age range, ASD diagnosis status, and gender.

Figure 9 panels A and B show that children in ASD had a higher G-causality from the right to the left brain hemisphere than controls. This scenario changed at approximately 14 years old. Figure 10 shows the boxplots of the Box–Cox transformed Wald’s test statistic obtained by the VAR method, separated by age range, ASD diagnosis status, and gender. According to a Welch’s *t*-test, there was a differential G-causality from the right to the left hemisphere in autistic subjects considering only females aged 6 to 13 years (p=0.014) and considering only males aged 16 to 60 years (p=0.009).

To verify the robustness of our approach, we reanalyzed the data using VAR orders four and six—the conclusions remain unchanged.

**Figure 11:**
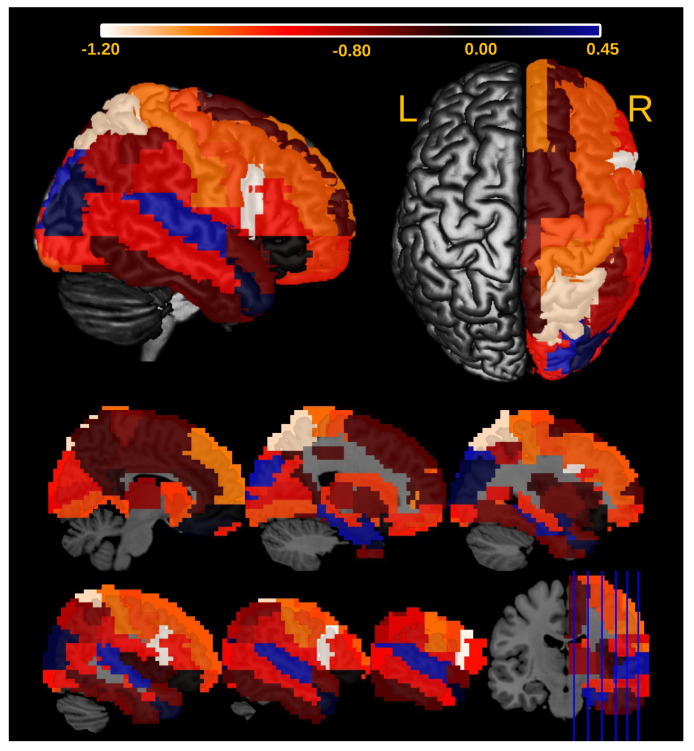
Heatmap describing the influence of the ROIs of the right brain on the significance of the interaction effect between AGE and ASD diagnosis ($\beta_{\mathrm{AGE}\times \mathrm{ASD}}$) in Equation (6). The more negative is the value (the effect of the *i*th ROI in the Granger causality as $t_{\mathrm{AGE}\times \mathrm{ASD}}-t_{\mathrm{AGE}\times \mathrm{ASD}}^{-i}$), the greater is the ROI’s influence. Notice that the pars opercularis and superior parietal gyrus are the ROIs that contribute the most to AGE × ASD.

We also identified the ROIs in the right hemisphere associated with the differential Granger causality between ASD and controls. To identify the ROIs, we did the following. We removed the *i*-th ROI (i=1,…,45) and re-ran the entire analysis. Let $t_{\mathrm{AGE}\times \mathrm{ASD}}$ be the t-value associated with the coefficient $\beta_{\mathrm{AGE}\times \mathrm{ASD}}$ in Equation (6) and $t_{\mathrm{AGE}\times \mathrm{ASD}}^{-i}$ be the t-value obtained in the analysis without the *i*-th ROI. Then, we could describe the effect of the *i*-th ROI in the Granger causality as $t_{\mathrm{AGE}\times \mathrm{ASD}}-t_{\mathrm{AGE}\times \mathrm{ASD}}^{-i}$. In Table 1, we see that $t_{\mathrm{AGE}\times \mathrm{ASD}}=-2.2948$. As a result, we identified two regions with the greatest impact on the significance of AGE×ASD interaction coefficient: pars opercularis and superior parietal gyrus (Figure 11).

Several studies [46] reported sex differences in ASD, which presents a greater prevalence in males and symptoms (repetitive and externalizing behaviors) [47]. Moreover, in previous neuroimaging studies, ref. [48] evaluated the male/female differences in functional connectivity during resting state to test whether they support the ‘neural masculinization’ hypothesis. The authors concluded that results pointed toward ASD as a disorder of sexual differentiation instead of masculinization in both genders. Moreover, ref. [49] found gender differences on the structural connectomes in ASD regarding white matter connectivity densities, suggesting that both structure and functions might be compromised. Complementary, it is well-established that ASD is a complex neurodevelopmental condition [50] with systems-level features evolving across the human lifespan [36]. In other words, brain abnormalities manifested in children with ASD are not the same at other developmental stages. Ref. [51] argue that there is current evidence from neuroimaging studies that sex differences in ASD are age-dependent. The authors concluded that studies should focus on large-sample studies and a lifespan perspective. In addition, Figure 11 depicts right pars opercularis as related to the AGE×ASD interaction effect, which is a novel contribution to the field. The majority of studies report the left opercularis as involved in language. Moreover, language functions are associated with both age and ASD. Thus, the involvement of the contralateral region is exciting but not unexpected. Furthermore, the involvement of the medial superior parietal gyrus is also of relevance because it is part of the Default Mode Network. This network is implicated in social cognition, which is impaired in ASD.

Future studies are necessary to understand the implications of these findings better. The current study illustrated functional network-based modeling using both a large sample and a lifespan approach. Remarkably, our findings are in line with previous studies highlighting differential sex and age-dependent effects of ASD on brain functioning compared to typical development subjects. Specifically, the interaction effects between AGE × ASD and AGE × SEX on interhemispheric functional connectivity is the main contribution of this illustration. Notably, the direction of our findings points toward a decrease in ASD effects with age and the latter with sex. The neurobiological mechanisms which explain these effects are still unknown, and many conjectures could be raised. Age cumulative environmental impacts from therapeutic interventions to coping strategies instruction may have a complex interaction with subjects’ genetic and neurodevelopmental features.

Further studies are necessary to unveil these dynamic mechanisms. We believe the field of systems biology may play a role. Thus, we advocate for developing novel analytical approaches to enhance our comprehension of these complex systems. For example, approaches focused on time-varying functional connectivity would complement our approach. Notice that our framework identifies Granger causality among networks based on the entire time series. In other words, it provides “an average” Granger causality from one network to another. However, Stramaglia et al. [52] proposed a way to identify local Granger causality. Their method offers a robust and computationally fast method to follow the information transfer and the time history of linear stochastic processes and nonlinear complex systems studied in the Gaussian approximation. They can identify Granger causality for each time point. On the other hand, they do not identify Granger causality among networks. We could combine their approach and ours to identify local Granger causality among networks time series as future work. Besides the work of Stramaglia et al. [52], there are other methods for time-varying connectivity inference. For a good review, refer to [53].

## 4. Conclusions

The development of novel analytical approaches is crucial to enhance our comprehension of Systems Biology. In the current study, we defined G-causality between graphs and proposed a framework to identify it, based on the combination of the concepts of spectral radius, random graphs, and the vector autoregressive model. Our computational simulations suggest that the proposed statistical test is adequate. In other words, we control the type I error while maintaining a considerable statistical power. Moreover, the illustration of our approach using the ABIDE I dataset provided new insights on brain connectivity disruptions in ASD patients and their relation to neurodevelopment and sex.

## Figures and Tables

**Table 1:** Results considering Wald’s test statistic as the response variable in Equation (6) for assessing G-causality from the right to the left hemisphere. We considered the VAR model with order p=5.

Parameter	Estimate	Std. Error	*t*-Value	*p*-Value
α	2.5270	0.2163	11.6795	<0.0001
$\beta_{\mathrm{FD}}$	−0.9295	0.8893	−1.0452	0.2963
$\beta_{\mathrm{SEX}}$	0.5956	0.2291	2.5997	0.0095
$\beta_{\mathrm{AGE}}$	0.0082	0.0073	1.1229	0.2619
$\beta_{\mathrm{ASD}}$	0.2945	0.1731	1.7013	0.0893
$\beta_{\mathrm{AGE}\times \mathrm{ASD}}$	−0.0204	0.0089	−2.2948	0.0220
$\beta_{\mathrm{SEX}\times \mathrm{AGE}}$	−0.0290	0.0126	−2.3036	0.0215

## Data Availability

The ABIDE I dataset is available at http://fcon_1000.projects.nitrc.org/indi/abide/. Source code and simulated data are available upon request.
